# Does the omission of vincristine in patients with diffuse large B cell lymphoma affect treatment outcome?

**DOI:** 10.1007/s00277-018-3437-z

**Published:** 2018-08-08

**Authors:** Charlott Mörth, Antonios Valachis, Amal Abu Sabaa, Daniel Molin, Max Flogegård, Gunilla Enblad

**Affiliations:** 1 Centre for Clinical Research Sörmland, Uppsala University, Uppsala, Sweden; 2 Department of Immunology, Genetics, and Pathology, Experimental and Clinical Oncology, Uppsala University, Uppsala, Sweden; 3 Department of Internal Medicine, Falun General Hospital, Falun, Sweden; 4 Cancercentrum, Mälarsjukhuset, 63188 Eskilstuna, Sweden

**Keywords:** Chemotherapy, Neurotoxicities, Hematology/oncology general, Non-Hodgkin lymphoma, Vincristine

## Abstract

The standard treatment for diffuse large B cell lymphoma (DLBCL) is rituximab with CHOP (cyclophosphamide, doxorubicin, vincristine (VCR), and prednisone). Maintaining high dose intensity of cytotoxic treatment has been associated with better outcome but little is known about the role of maintaining VCR. This study aimed to answer whether the omission of vincristine due to neurotoxicity affects patient outcome. A Swedish cohort of patients primarily treated with curative intent for DLBCL or high-grade malignant B cell lymphoma was retrospectively analyzed. In total, 541 patients treated between 2000 and 2013 were included. Omission of VCR was decided in 95 (17.6%) patients and was more often decided during the last three cycles (*n* = 86, 90.5%). The omission of VCR did not affect disease-free or overall survival neither in the whole cohort nor in elderly patients. On the contrary, the relative dose intensity of doxorubicin was associated with overall survival (*p* = 0.014). Kidney or adrenal involvement (*p* = 0.014) as well as bulky disease (*p* = 0.037) was found to be associated with worse overall survival. According to our results, clinicians can safely decide to omit VCR in case of severe neurotoxicity due to VCR but should be aware of the importance of giving adequate doses of doxorubicin during treatment given the growing body of evidence on the role of dose intensity on survival. Considering the association of bulky disease and kidney/adrenal manifestation of lymphoma on survival, further studies should focus on whether the treatment options for these subgroups need to be individualized.

## Introduction

Diffuse large B cell lymphoma (DLBCL) is the most common subtype of lymphoma accounting for up to 40% of all cases [1]. The standard of care for DLBCL is a combination of rituximab (R) with CHOP (cyclophosphamide (CPM), doxorubicin (DXR), vincristine (VCR), and prednisone) [2, 3].

Maintaining high relative dose intensity (RDI) of CHOP, with or without R, has been associated with better progression-free survival (PFS) and overall survival (OS) in DLBCL [4–8]. However, the occurrence of dose-limiting toxicity with each of these chemotherapeutic agents can compromise the RDI and, as a result, the treatment outcome. The most common dose-limiting toxicity for CPM and DXR that can lead to lower RDI is neutropenia/febrile neutropenia [9]. For this type of toxicity, primary prophylaxis with granulocyte-colony-stimulating factor is a well-established strategy to decrease the risk and severity of neutropenia and maintain RDI and is recommended from current international guidelines [10].

On the other hand, the dose-limiting toxicity of VCR is neurotoxicity [11–13] which is more complicated in terms of maintaining RDI since there are no prophylactic strategies, only dose reduction or treatment omission in patients presenting with signs/symptoms of neurotoxicity.

Only one study specifically investigated the role of RDI of VCR in R-CHOP treatment and found, in a relatively small cohort of 86 patients, that lower RDI of VCR was associated with decreased survival despite high RDI of CPM and DXR [7].

However, the small sample size of the study and the single-center data make the generalizability of the results questionable.

As a result, the clinical question on whether the omission of VCR from one or more cycles of therapy could jeopardize the survival in patients with DLBCL has not yet been adequately addressed.

The purpose of this study was to investigate the effect of VCR omission in patients with DLBCL or high-grade malignant B cell lymphoma treated with R-CHOP on disease-free survival (DFS) and overall survival (OS).

## Patients and methods

### Study design and setting

This was a Swedish multi-institutional retrospective cohort study including all patients diagnosed with DLBCL or subgroups of high-grade malignant B cell lymphoma between 2000 and 2013 in four different institutions: General Hospital of Eskilstuna Mälarsjukhuset (Eskilstuna), Uppsala University Hospital (Uppsala), Falu General Hospital (Falun), and Gävle General Hospital (Gävle).

### Patient population

Adult patients (> 18 years old) registered in the National Swedish Lymphoma Registry (Informationsnätverk för Cancervården, INCA) who were primary treated as DLBCL with at least one course of rituximab with either CHOP, CHOEP (CHOP plus etoposide), or mini-CHOP (reduced-dose CHOP) were identified. Patients with primary mediastinal and testicular lymphoma were included as well as patients with follicular lymphoma transformed to DLBCL if the only prior treatment given for follicular lymphoma was radiotherapy.

We excluded patients with primary central nervous system lymphoma and Burkitt lymphoma as well as patients that were considered by the treating physician as too frail to receive any treatment or treated with less intensive chemotherapy with no intention to cure or combinations other than CHOP or CHOEP due to comorbidities (i.e., CEOP, liposomal doxorubicin—COP, bendamustine).

The study was approved by the local review board in Uppsala, Sweden (Dnr 2014/233).

### Data collection—definitions

Patient charts and medical records from the in-hospital computer-based medical records were used to extract demographic, clinical, biochemical, and pathologic data relevant to the study.

The following data were recorded: age at diagnosis, gender, area of residency, body mass index (BMI), type of lymphoma, date of diagnosis (defined as date of biopsy, either needle or surgical), presence of any autoimmune disease (AI), presence of B-symptoms (fever > 38 °C, drenching night sweats, unintentional weight loss of > 10% of body weight over a period of < 6 months) at diagnosis, performance status (PS 0–4) according to Eastern Cooperative Oncology Group (ECOG), stage at diagnosis (Ann Arbor I–IV), International Prognostic Index (IPI) score, lactate dehydrogenase (LDH) (above upper limit normal (ULN) or normal), bulky disease (defined as tumor with a diameter of > 7.5 cm in transverse diameter), disease in > 1 extranodal organ, occurrence of kidney or adrenal involvement of disease, type of treatment, doses of DXR and VCR, occurrence of VCR omission, treatment outcome at the end of treatment (complete response (CR), partial response (PR), stable disease (SD) or progressive disease (PD), defined according to International Response Criteria [14]), date of relapse, date of death, cause of death (determined by information in charts or in death certificates), and date of last follow-up.

All histological diagnostics were made by the local or regional departments of pathology for each hospital using definitions of the 2008 WHO—classification of lymphoma [15].

Relative dose intensity (RDI) for DXR (DoxoRDI) was calculated according to Yamagushi et al. [8]. DFS was defined as the time between diagnosis and last follow-up in the absence of relapse. If a relapse occurred, DFS was set to time from diagnosis to date of relapse (date of clinical/radiation finding or biopsy). If the patient never reached CR or PR and subsequently died from lymphoma, DFS time was set to zero. Overall survival (OS) was defined as time from diagnosis to the date of death from any cause. Patients that were alive to the date of last follow-up were censored.

### Statistical analysis

Categorical variables were expressed as number (%) and continuous variables as median (range). For bivariate comparisons, the chi-square test was used for categorical variables whereas the *t* test or (for non-normally distributed variables) the non-parametric Mann-Whitney test was used for continuous variables.

Time-to-event outcomes (DFS and OS) were analyzed by using the Kaplan-Meier and the log-rank test was used to test statistical significance. A two-sided *p* value of ≤ 0.05 was regarded as cutoff for statistical significant results in comparisons between groups.

Any variables significantly associated with DFS or OS in bivariate analyses (with a *p* value of ≤ 0.05) were considered for entry into a multivariate Cox proportional hazards regression analysis. Two separate multivariate analyses were performed for DFS and OS, respectively. Omission of VCR was included in both models as an independent variable of interest.

The main analyses were performed by using the complete case analysis approach to handle missing data. A sensitivity analysis was performed using the multiple imputation (MI) method. The rates of missing values from potential predictors for DFS or OS ranged from 0 to 27%. We decided a priori to exclude variables with > 30% missing values. Missing data were imputed for the following variables (missing values are presented in parentheses): extranodal engagement of disease (20.1%), kidney or adrenal involvement (20.1%), LDH level (20.9%), PS (23.8%), bulky disease (24.2%), and BMI (27%). The imputation was performed using the chained equations method and 10 multiple imputed datasets were created and used for the analyses.

Two subgroup analyses were performed. First, we performed a subgroup analysis restricted to patients ≥ 70 years old considering that older age has been associated with higher risk for reduced RDI [4]. We performed an additional subgroup analysis to investigate a dose-dependent relationship between cycle of VCR omission and treatment outcome. Specifically, we calculated, using Cox proportional-hazards model, the adjusted hazard ratio for DFS and OS based on chemotherapy cycle number in which VCR was omitted (cycles 1–3, cycle 4, cycle 5, cycle 6) compared to no omission of VCR.

Statistical analyses were performed with the IBM statistics SPSS version 22.

## Results

### Study cohort

In total, 541 patients were considered eligible for the study. Baseline characteristics are presented in Table 1.

**Table 1:** Baseline characteristics

	VCR omission (%)	VCR full dose (%)	*p* value
Number of pts	95	446	
Demographics			
Age, median (18–91)	66.0 (50–84)	66.0 (18–91)	0.996
< 60	15 (15.8)	139 (31.2)	0.003
≥ 60	80 (84.2)	307 (68.8)	
Sex			
Male	58 (61.1)	256 (57.4)	0.512
Female	37 (38.9)	190 (42.6)	
PS			
0–1	81 (85.3)	281 (63.0)	0.376
2–4	14 (14.7)	36 (8.1)	
Missing	0	129 (28.9)	
Stage			
1–2	29 (30.5)	158 (35.4)	0.354
3–4	66 (69.5)	287 (64.4)	
Missing	0	1 (0.2)	
IPI			
0–2	38 (40.0)	234 (52.5)	0.006
3–5	57 (60.0)	186 (41.7)	
Missing	0	26 (5.8)	
LDH			
> ULN	65 (68.4)	216 (48.4)	0.520
≤ ULN	30 (31.6)	117 (26.2)	
Missing	0	113 (25.3)	
Bulky^a^			
Yes	20 (21.1)	73 (16.3)	0.665
No	75 (78.9)	242 (54.3)	
Missing	0	131 (29.4)	
AI			
Yes	18 (18.9)	82 (18.4)	0.898
No	77 (81.1)	364 (81.6)	
Extranodal^b^			
> 1	30 (31.6)	60 (13.5)	0.004
≤ 1	65 (68.4)	277 (62.1)	
Missing	0	109 (24.4)	
Kidney/adrenal^c^			
Yes	3 (3.2)	18 (4.1)	0.382
No	92 (96.8)	319 (71.5)	
Missing	0	109 (24.4)	
BMI, median (16.2–44.10)	25.7 (16.2–41.4)	25.8 (16.9–44.1)	0.850
Missing	3 (3.2)	300 (67.3)	
Treatment			
CHOP	79 (83.2)	378 (84.8)	0.697
CHOEP	16 (16.8)	68 (15.2)	
DoxoRDI			0.396
≤ 70%	6 (6.3)	28 (6.3)	
> 70%	83 (87.4)	261 (58.5)	
Missing	6 (6.3)	157 (35.2)	

*Pts*, patients; *PS*, performance status; *IPI*, International Prognostic Index; *LDH*, lactate dehydrogenase; *ULN*, upper limit normal; *AI*, autoimmune disease; *BMI*, body mass index; *DoxoRDI*, doxorubicin dose intensity

^a^Tumor mass > 7.5 cm

^b^Involvement of extranodal organ

^c^Kidney or adrenal involvement

In 95 (17.6%) patients, VCR was omitted due to toxicity. Omission was more often decided during the last three cycles (*n* = 86, 90.5%). Patients with VCR omission were older (*p* value = 0.003) with higher IPI (*p* value = 0.006) and higher amount of ≥ 1 extranodal involvement (*p* value = 0.003) compared with patients that received all the planned doses of VCR.

### Prognostic factors for DFS and OS

**Fig. 1:**
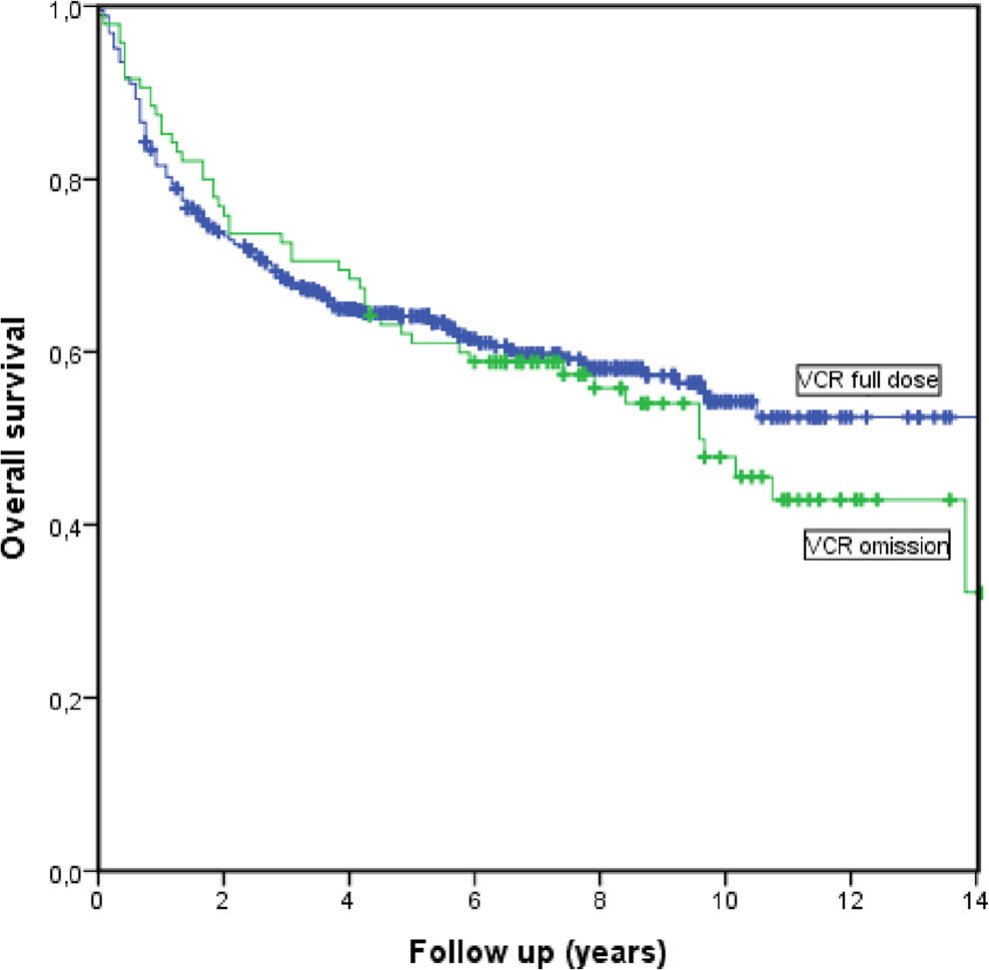
Kaplan-Meier for survival comparing full dose VCR vs omission of VCR. *p* = 0.572

Bivariate analysis revealed nine predictors possibly associated with DFS: PS ≥ 2, stage III–IV, IPI ≥ 3, increased LD, presence of bulky disease, extranodal involvement, kidney/adrenal involvement, BMI ≥ 25, and DoxoRDI ≤ 70%; 10 predictors were associated with OS: older age, PS ≥ 2, stage III–IV, IPI ≥ 3, increased LD, presence of bulky disease, kidney/adrenal involvement, BMI ≥ 25, DoxoRDI ≤ 70%, and chemotherapy used. Omission of VCR was not associated with worse OS (Fig. 1).

The results of the multivariate Cox regression analyses for DFS and OS are shown in Table 2. For DFS, only advanced stage at diagnosis was found to be significantly associated with worse outcome (HR: 2.04, 95% CI (confidence interval) 1.01–4.00). In respect of OS, kidney/adrenal involvement (HR 2.45, 95% CI 1.20–4.98), DoxoRDI ≤ 70% (HR 2.04, 95% CI 1.15–3.61), age ≥ 60 years old (HR 1.94, 95% CI 1.09–3.48), and bulky disease (HR 1.58, 95% CI 1.03–2.42) were significantly associated with worse survival.

**Table 2:** Multivariable Cox regression analysis of disease-free survival (DFS) and overall survival (OS)

	DFS		OS	
	Hazard ratio (95% CI)	*p* value	Hazard ratio (95% CI)	*p* value
Age ≥ 60	Not included		1.94 (1.09–3.48)	0.025
Treatment^a^	Not included		1.76 (0.90–3.43)	0.096
PS ≥ 2	1.62 (0.74–3.57)	0.235	1.77 (0.84–3.74)	0.134
Stage > 2	2.04 (1.01–4.00)	0.047	1.59 (0.88–2.88)	0.127
IPI > 2	1.33 (0.70–2.50)	0.385	1.14 (0.60–2.16)	0.686
LDH > ULN	1.09 (0.63–1.89)	0.778	1.03 (0.63–1.69)	0.893
Bulky^b^	1.30 (0.81–2.10)	0.283	1.58 (1.03–2.42)	0.037
Oncovin omission^c^	1.21 (0.76–1.95)	0.421	1.13 (0.75–1.71)	0.571
Extranodal^d^ > 1	1.02 (0.59–1.78)	0.932	Not included	
Kidney/adrenal^e^	1.72 (0.78–3.85)	0.171	2.45 (1.20–4.98)	0.014
BMI ≥ 25	0.89 (0.58–1.37)	0.591	0.98 (0.67–1.43)	0.904
DoxoRDI ≤ 70%	1.88 (0.97–3.67)	0.063	2.04 (1.15–3.61)	0.014

*CI*, confidence interval; *PS*, performance status; *IPI* International Prognostic Index; *LDH*, lactate dehydrogenase; *ULN*, upper limit of normal; *BMI*, body mass index; *DoxoRDI*, doxorubicin relative dose intensity

^a^CHOP vs. CHOEP

^b^Tumor mass > 7.5 cm

^c^Omission of Oncovin at any course

^d^Involvement of extranodal organ

^e^Kidney or adrenal involvement

### Omission of VCR and treatment outcome

Omission of VCR was not associated with either DFS or OS in multivariate analyses (HR for PFS 1.21, 95% CI 0.76–1.95; HR for OS 1.13, 95% CI 0.75–1.71).

In the sensitivity analysis using the MI method to handle missing values, the lack of association between omission of VCR and DFS (HR 1.20, 95% CI 0.81–1.78) or OS (HR 1.06, 95% CI 0.76–1.48) remained unchanged and non-significant.

**Fig. 2:**
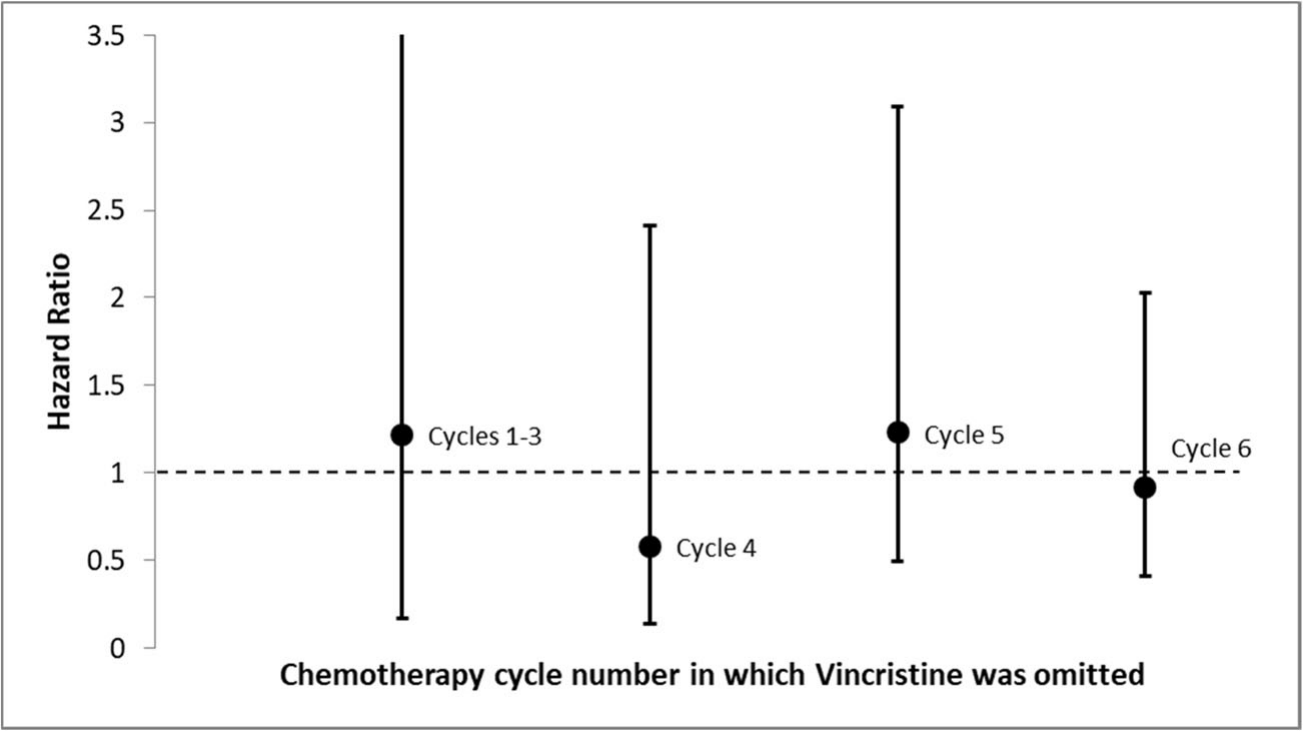
Hazard ratio (OS) for omission of VCR in cycles 1–3, 4, 5, and 6 vs no omission

The lack of association between VCR omission and survival was evident irrespective of the number of the cycle in which VCR was omitted (Fig. 2). Compared to patients treated with six VCR cycles, those who received only one to three cycles showed comparable survival (HR for DFS 0.30, 95% CI 0.07–1.30; HR for OS 1.22, 95% CI 0.17–9.01).

When the analysis was restricted to patients ≥ 70 years old (*n* = 204), the omission of VCR was not found to be associated to survival either (HR for DFS 1.43, 95% CI 0.53–3.83; HR for OS 1.30, 95% CI 0.53–3.16).

## Discussion

The present study, using a large cohort of consecutive patients, could not find that omission of VCR affects prognosis of DLBCL in terms of DFS or OS. The lack of association between omission of VCR and prognosis remained unchanged when analysis was restricted to the elderly population. In addition, the timing of omission showed no correlation to prognosis either, namely there was no difference whether VCR was omitted early or late in the treatment course.

The potential effect of reduced VCR dose on DLBCL prognosis was previously investigated only in one study. Utsu et al. included 86 patients treated with R-CHOP-21 due to DLBCL and found that survival rate was lower for RDI VCR < 85% despite adequate CPM and DXR doses. However, we could not confirm these results in our study cohort. Several possible explanations for these contradictory results can be speculated. First, the study cohorts differ between the two studies. Utsu et al. included only cases treated with R-CHOP-21 due to DLBCL whereas we used more wide inclusion criteria and included patients with more diverse chemotherapy regimens in an effort to better reflect the daily clinical practice. Furthermore, we used a more pragmatic and clinically relevant approach for reduced VCR dose, namely the omission of VCR at a specific treatment course instead of RDI which is more complicated to interpret. In addition, the reason for VCR dose reduction in Utsu et al.’s study was not captured whereas the reason for VCR omission in our cohort was neurotoxicity in all cases. Considering the fact that our study cohort is considerably larger and seems to be more representative of the daily clinical practice, our results may be more robust.

The incidence and severity of VCR neurotoxicity are correlated with the treatment duration and dosing but there is a considerable variability among patients in the pharmacokinetics of VCR and no absolute correlation between neurotoxicity and plasma levels of VCR has been observed [12, 16]. As a result, it is difficult to correlate VCR dose to treatment effect. A potential explanation for our findings of the lack of association between VCR omission, even early during the treatment course, and survival could be that the presence of neurotoxicity is correlated to higher intra-cellular VCR bioavailability and as so might be associated with a better response to treatment.

Our results on the positive association between maintaining high RDI for DXR and treatment outcome are in accordance with prior studies [4–8] and suggest that DXR might be a more important chemotherapeutic agent than VCR in the treatment of DLBCL.

In our cohort, we found a negative correlation between bulky disease as well as kidney/adrenal involvement and OS. Our findings are in accordance with prior studies. Specifically, bulky disease has been associated with worse outcome [17], probably due to a lower cytotoxic dose in the central, poorly vascularized parts of the tumor. The use of consolidation radiotherapy after Rituximab-chemotherapy seems to offer some benefit in those patients [18, 19]. In our cohort, only few patients received consolidation radiotherapy as most of the patients received treatment before the recommendation of consolidation radiotherapy for bulky disease was included in the national treatment guidelines for DLBCL. The association of kidney/adrenal involvement and worse outcome has also been observed in prior studies, mainly due to the fact that kidney/adrenal involvement is a risk factor for central nervous system (CNS) relapse [20–24]. Whether the association of kidney/adrenal involvement and worse outcome that we observed is due to CNS relapse or due to early relapse in general is uncertain because we did not perform separate analyses for CNS relapse. The mechanism of lymphoma dissemination into the CNS due to kidney/adrenal involvement is unclear but molecular characteristics of extranodal lymphoma cells as well as tumor microenvironment might be the key elements to facilitate dissemination to the CNS.

There are several limitations on this study that need to be discussed. First, the retrospective nature of the study is prone to well-described bias. Second, there were missing values in some variables; however, our results remained stable even when we dealt with missing values by using MI methodology. In addition, unlike other studies investigating RDI and treatment outcome, the RDI CPM was not analyzed. Finally, the number of cases was limited for calculation of the effect of omission of VCR after the first, second, or third treatment course separately.

In conclusion, the omission of VCR does not affect either DFS or OS in patients with DLBCL treated with R-CHOP/CHOEP/mini-CHOP. As a result, clinicians can safely decide to omit VCR in case of severe neurotoxicity due to VCR. Considering the association of bulky disease and kidney/adrenal manifestation of lymphoma on survival, further studies should focus on whether the treatment options for these subgroups need to be individualized. Finally, clinicians should be aware of the importance of giving adequate doses of DXR during treatment given the growing body of evidence on the role of dose intensity on survival.
